# Performance of artificial intelligence for biventricular cardiovascular magnetic resonance volumetric analysis in the clinical setting

**DOI:** 10.1007/s10554-022-02649-1

**Published:** 2022-06-29

**Authors:** Suzan Hatipoglu, Raad H. Mohiaddin, Peter Gatehouse, Francisco Alpendurada, A. John Baksi, Cemil Izgi, Sanjay K. Prasad, Dudley J. Pennell, Sylvia Krupickova

**Affiliations:** 1 CMR Unit, Royal Brompton Hospital, Guy’s and St Thomas’s NHS Foundation Trust, Sydney Street, London, SW3 6NP UK; 2 Cardiology Department, Kettering General Hospital, Kettering, UK; 3 National Heart & Lung Institute, Imperial College, London, UK; 4 Pediatric Cardiology Department, Royal Brompton Hospital, Guy’s and St Thomas’s NHS Foundation Trust, London, UK

**Keywords:** Cardiovascular magnetic resonance, Artificial intelligence, Myocardial disease, Ventricular function, Ventricular volumes, Ejection fraction, Myocardial segmentation

## Abstract

Cardiovascular magnetic resonance (CMR) derived ventricular volumes and function guide clinical decision-making for various cardiac pathologies. We aimed to evaluate the efficiency and clinical applicability of a commercially available artificial intelligence (AI) method for performing biventricular volumetric analysis. Three-hundred CMR studies (100 with normal CMR findings, 50 dilated cardiomyopathy, 50 hypertrophic cardiomyopathy, 50 ischaemic heart disease and 50 congenital or valvular heart disease) were randomly selected from database. Manual biventricular volumetric analysis (CMRtools) results were derived from clinical reports and automated volumetric analyses were performed using short axis volumetry AI function of CircleCVI^42^ v5.12 software. For 20 studies, a combined method of manually adjusted AI contours was tested and all three methods were timed. Clinicians` confidence in AI method was assessed using an online survey. Although agreement was better for left ventricle than right ventricle, AI analysis results were comparable to manual method. Manual adjustment of AI contours further improved agreement: within subject coefficient of variation decreased from 5.0% to 4.5% for left ventricular ejection fraction (EF) and from 9.9% to 7.1% for right ventricular EF. Twenty manual analyses were performed in 250 min 12 s whereas same task took 5 min 48 s using AI method. Clinicians were open to adopt AI but concerns about accuracy and validity were raised. The AI method provides clinically valid outcomes and saves significant time. To address concerns raised by survey participants and overcome shortcomings of the automated myocardial segmentation, visual assessment of contours and performing manual corrections where necessary appears to be a practical approach.

**Supplementary Information:**

The online version contains supplementary material available at 10.1007/s10554-022-02649-1.

## Introduction

A huge amount of healthcare data is generated by diagnostic imaging; however, it is challenging to find a skilled workforce for the analysis [1]. Artificial intelligence (AI) methods have been developed to address this problem and they proved to be applicable especially for medical imaging analysis [2]. A lack of understanding of how AI algorithm processes the data is less concerning as the accuracy of the analysis can be visually inspected [3]. The routine clinical use of AI applications has the potential to save clinicians’ time from tasks that need specific pattern recognition but are also repetitive [4]. Implementation of AI into practice is a real-life challenge and limitations should be addressed [5]. Trust in AI diagnostics and user experience are important hurdles for routine clinical use (6).

Biventricular volumetric analysis provides key information for the diagnosis and follow up of many cardiac conditions [7]. Cardiovascular magnetic resonance (CMR) is the gold standard method to perform these measurements, but the analysis takes considerable time with repetitive contouring of cardiac structures, a process called “myocardial segmentation”. The most used AI method in CMR volumetric analysis is deep learning with convolutional neural networks (CNN) [8]. AI applications for CMR volumetric analysis provided satisfactory results and acceptable agreement when compared to manual analysis by human controls in some recent studies [9–12]. However, these studies were performed in highly controlled research settings with optimal image quality and did not include diverse pathological cardiac conditions [13–15]. The reliability and efficiency of AI in routine clinical practice has not been tested in randomized controlled trials. Commercially available image analysis software packages introduced CNN-based automated image segmentation, however there is no convincing literature to support use of AI segmentation output interchangeably with manual analysis [8]. To establish trust, testing of AI performance in real-life clinical situations could be an effective apprach for implementation [5].

This work aimed to evaluate the performance, clinical applicability and the potential for time saving of commercially available AI module of Circle CVI^42^ CMR analysis software version 5.12 for biventricular volumetric analysis from short axis cine images.

## Methods

**Fig. 1:**
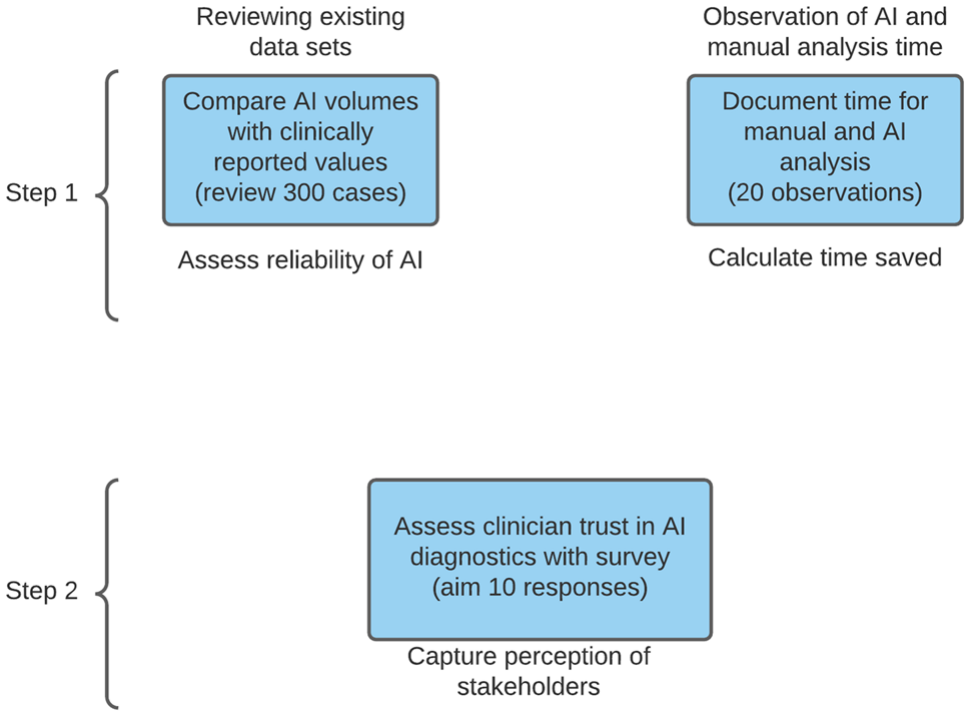
Study protocol using combination of three data collection methods. AI: artificial intelligence

Three hundred randomly selected clinical CMR image datasets (scans performed between 11/2009 and 04/2021) were reanalysed with the AI method (Circle CVI^42^ CMR analysis software version 5.12, Calgary, Canada) and the output from fully automated LV and RV volumetric analysis was recorded. Manual analysis results (CMRTools, Cardiovascular Imaging Solutions, London, UK) were derived from clinical reports and agreement with AI output was tested. To test AI performance in different disease conditions, 100 cases referred to exclude cardiac disease but with a normal scan, 50 cases with dilated cardiomyopathy, 50 cases with hypertrophic cardiomyopathy, 50 cases with ischaemic heart disease and 50 cases with valvular or congenital heart disease were included. A further 20 studies from the normal range subcategory were randomly selected for the AI contours to be manually adjusted by an experienced CMR clinician where necessary. These 20 studies were also reanalysed manually by a single expert operator using CVI^42^ software to assess difference between manual analysis using different vendors (CMRTools used for clinical reporting) and effect of multiple operators analysing clinical scans. Studies mentioning suboptimal image quality in the clinical reports were excluded. Manual and AI analysis were timed with a stopwatch for 20 studies to calculate efficiency benefit. Finally, user trust in the AI method was assessed in a survey which also revealed the results of the agreement analysis. Surveys were conducted via Qualtrics link e-mailed to participants. The survey took approximately 5 min to complete (survey questions are presented in online Appendix 1). Study protocol is summarised in Fig. 1.

This was a retrospective analysis of data collected for routine clinical care. The study was registered and approved by the Royal Brompton Hospital Safety and Quality Department (approval number 004426) and individual informed consent was not required in line with UK National Research Ethics Service guidance.

### CMR scanning protocol, volumetric analysis, and image quality

The CMR scans were performed for clinical indications on several scanners with conventional ECG gating and array coils at 1.5 T (Magnetom Aera and Magnetom Avanto^fit^ Siemens Healthineers). Long axis and stack of short axis cines were acquired with bSSFP as described in the literature for a standard clinical CMR study [16, 17]. In line with departmental standards of practice, left and right ventricular (RV) volumes, ejection fraction (EF), and left ventricular (LV) mass were calculated using the short­axis cine stack and indexed to body surface area (BSA). Papillary muscles and LV/RV trabeculations were included in the myocardial mass calculation and excluded from the blood volume. Volumes were indexed to body surface area (BSA) calculated using the Mosteller formula [16, 17]. Manual volumetric analysis data were derived from clinical reports.

The image quality of the standard short axis cine stack was assessed as described in the published EuroCMR registry criteria [18]. According to these criteria, 1 point was given if an artefact impeded the visualization of more than one-third of the LV endocardial border at end-systole and/or diastole on a single short-axis slice. If the artefact involved 2 or 3 slices, 2 or 3 points were given, respectively. In terms of LV coverage 2 points were given if the apex was not covered and 3 points if a basal slice or more than one slice in the stack were missing. An image quality score of 0 corresponded to a study with no significant artefact affecting the clinical evaluation, no missing or unusable slices and optimal orientation of the stack.

Accuracy of AI myocardial segmentation was visually assessed on each short axis cine slice and qualitatively scored using one of three categories defined as “good” if no manual correction of AI contours was needed, “adequate” in cases where minimal changes were needed at the base of the heart usually involving the valve planes, or “suboptimal” if several slices of AI analysis necessitated manual modification to be deemed clinically acceptable. Additionally, for 20 consecutive AI analyses in the normal subgroup, manual adjustment of AI contours was performed. Improvement in agreement with this combination of AI and manual methods was evaluated.

## Statistical analysis

Quantitative data obtained were analysed using IBM SPSS Statistics Software Version 27 (International Business Machines, Armonk, New York, USA) and MedCalc® Statistical Software version 20.015 (MedCalc Software Ltd, Ostend, Belgium; https://www.medcalc.org; 2021) was used to generate Bland–Altman plots. Normal distribution was tested with the Shapiro–Wilk test. Normally distributed parameters were presented as mean ± SD, whereas parameters not meeting normality were presented as median (interquartile range). Dependent variables were compared using the Wilcoxon signed-rank test. Agreement between manual and AI analysis output was tested using intra-class correlation coefficients (ICC) based on a model of absolute agreement, considered excellent if ICC > 0.8, good between 0.6 and 0.79, fair between 0.4 and 0.59 and poor below 0.4 [19], 95% confidence intervals were also reported [19]. Bland–Altman plots were used to assess the combined (AI with manual adjustment of contours) method. Within-subject coefficient of variation (CoV) was calculated as SD of the differences divided by the mean. The Kruskal–Wallis H test was used to assess impact of image quality score on agreement. All tests were 2 tailed, and p < 0.05 was considered statistically significant. Qualitative data obtained from survey was presented descriptively and reported using the summary provided by Qualtrics (2021).

## Results

The selected CMR studies included 185 males (61.7%) and 115 females with median age of 50 (28) years. CMR indications, study image quality and scoring of AI myocardial segmentation data are presented using previously described subcategories in Table 1. Prospective gating was used in 48 studies (16%) to troubleshoot arrhythmia related image degradation and routine retrospectively gated acquisition was applied for the remaining studies.

**Table 1:** Demographic characteristics of the study population

*All studies, n* = *300*	
Gender, n	115 females, 185 males
Age, years	50, (28)
BSA, m^2^	1.89 ± 0.25
CMR findings	
No pathology, n	100
Dilated cardiomyopathy, n	50
Hypertrophic cardiomyopathy, n	50
Ischaemic heart disease, n	50
Valvular or congenital disease, n	50
Image quality	
Score 0 (excellent), n	39
Score 1 (good), n	213
Score 2 (adequate), n	48
AI contours score	
Good, n	30
Adequate, n	220
Suboptimal, n	50
*CMR studies with normal findings, n* = *100*	
Gender, n	53 females, 47 males
Age, years	43, (27)
BSA, m^2^	1.82 ± 0.18
CMR indication	
Cardiomyopathy screen, n	64
Ischaemia assessment, n	13
Suspected myocarditis, n	11
Arrhythmia, n	9
Aorta assessment, n	3
Image quality	
Score 0 (excellent), n	31
Score 1 (good), n	55
Score 2 (adequate), n	14
AI contours score	
Good, n	24
Adequate, n	67
Suboptimal, n	9
*Dilated cardiomyopathy studies, n* = *50*	
Gender, n	8 females, 42 males
Age, years	50, (38)
BSA, m^2^	2.05, (0.23)
Image quality	
Score 0 (excellent), n	None
Score 1 (good), n	37
Score 2 (adequate), n	13
AI contours score	
Good, n	1
Adequate, n	36
Suboptimal, n	13
*Hypertrophic cardiomyopathy studies, n* = *50*	
Gender, n	14 females, 36 males
Age, years	60, (22)
BSA, m^2^	1.87 ± 0.20
Image quality	
Score 0 (excellent), n	None
Score 1 (good), n	46
Score 2 (adequate), n	4
AI contours score	
Good, n	3
Adequate, n	45
Suboptimal, n	2
*Ischaemic heart disease cases, n* = *50*	
Gender, n	15 females, 35 males
Age, years	66.7 ± 11.3
BSA, m^2^	1.95 ± 0.26
Image quality	
Score 0 (excellent), n	2
Score 1 (good), n	39
Score 2 (adequate), n	9
AI contours score	
Good, n	None
Adequate, n	41
Suboptimal, n	9
*Valvular heart disease or congenital cases, n* = *50*	
Gender, n	25 females, 25 males
Age, years	38, (23)
BSA, m^2^	1.83 ± 0.24
CMR indication	
Aortic valve disease, n	11
Mitral valve disease, n	3
Pulmonary valve disease, n	3
Shunt lesions, n	6
Repaired Tetralogy of Fallot, n	14
TGA after arterial switch, n	1
Coarctation of aorta, n	12
Image quality	
Score 0 (excellent), n	6
Score 1 (good), n	36
Score 2 (adequate), n	8
AI contours score	
Good, n	2
Adequate, n	31
Suboptimal, n	17

Normal distribution was tested with Shapiro–Wilk test. Normally distributed parameters presented as mean ± SD and parameters not meeting normality presented as median, (interquartile range)

*AI* artificial intelligence; *BSA* body surface area; *CMR* cardiovascular magnetic resonance; *TGA* transposition of great arteries

In the overall study cohort, agreement between manual and automated AI analysis was excellent for LV parameters [ICC 0.946 (95% CI, 0.932–0.958) for LV EF] and good for RV parameters, ICC 0.784 (95% CI, 0.127–0.913) for RV EF. For all groups, indexed end-diastolic volumes (EDVi) were highly reproducible with AI, ICC for LV EDVi 0.959 (95% CI, 0.740–0.985) and RV EDVi 0.918 (95% CI, 0.896–0.934). The highest within subject CoVs were observed for end-systolic volume indices (ESVi) -10.9% for LV ESVi and 16.6% for RV ESVi- and RV EF (13.1%). The agreement trends and scores were reproducible across subgroups with different cardiac pathologies. LV EDVi, LV ESVi, LV EF and RV EF were frequently underestimated, whereas LV mass index, RV EDVi and RV ESVi were usually overestimated by the AI method, see Table 2 for detailed agreement statistics.

**Table 2:** Agreement between manual and fully automated AI volumetric analysis

	Manual analysis	AI analysis	% CV	P value*	ICC
*Agreement for all studies, n* = *300*					
LV EDVi, mL/m^2^	86.7 ± 30.2	78.6 ± 27.0	7.6	< 0.001	0.959
LV ESVi, mL/m^2^	36.5 ± 30.2	33.6 ± 27.1	10.9	< 0.001	0.980
LV EF, %	61.4 ± 14.2	60.2 ± 14.9	6.5	0.073	0.946
LV mass index, g/m^2^	71.1 ± 22.3	75.4 ± 22.5	8.0	< 0.001	0.936
RV EDVi, mL/m^2^	83.3 ± 21.4	84.4 ± 20.7	7.5	0.001	0.918
RV ESVi, mL/m^2^	35.6 ± 17.8	42.1 ± 16.4	16.6	< 0.001	0.896
RV EF, %	58.4 ± 10.0	50.5 ± 12.4	13.1	< 0.001	0.784
*CMR studies with normal volumetric analysis findings, n* = *100*					
LV EDVi, mL/m^2^	79.6 ± 11.7	72.3 ± 11.2	7.2	< 0.001	0.854
LV ESVi, mL/m^2^	27.6 ± 6.7	25.9 ± 7.2	10.4	0.001	0.817
LV EF, %	65.6 ± 5.3	64.2 ± 8.3	5.4	0.484	0.640
LV mass index, g/m^2^	59.1 ± 11.9	63.8 ± 11.7	7.3	< 0.001	0.895
RV EDVi, mL/m^2^	81.7 ± 14.3	86.1 ± 15.3	6.8	< 0.001	0.877
RV ESVi, mL/m^2^	32.1 ± 8.4	39.4 ± 9.3	16.2	< 0.001	0.738
RV EF, %	61.0 ± 6.0	54.5 ± 5.6	8.9	< 0.001	0.507
*CMR studies with dilated cardiomyopathy diagnosis, n = 50*					
LV EDVi, mL/m^2^	121.8 ± 50.7	107.6 ± 47.2	9.4	< 0.001	0.967
LV ESVi, mL/m^2^	72.2 ± 50.5	67.6 ± 47.3	8.5	< 0.001	0.975
LV EF, %	73.4 ± 7.7	71.7 ± 9.1	10.9	0.739	0.861
LV mass index, g/m^2^	82.9 ± 23.9	90.8 ± 31.4	9.0	0.001	0.901
RV EDVi, mL/m^2^	93.2 ± 30.1	90.8 ± 28.2	5.9	0.858	0.925
RV ESVi, mL/m^2^	52 ± 27.3	57.2 ± 21.0	15.0	< 0.001	0.892
RV EF, %	46.0 ± 12.3	36.0 ± 14.8	24.6	< 0.001	0.694
*CMR studies with hypertrophic cardiomyopathy diagnosis, n = 50*					
LV EDVi, mL/m^2^	72.2 ± 10.8	69.7 ± 10.5	5.2	0.030	0.875
LV ESVi, mL/m^2^	19.3 ± 7.0	19.8 ± 7.9	12.7	0.671	0.923
LV EF, %	73.4 ± 7.7	71.7 ± 9.1	4.9	0.480	0.861
LV mass index, g/m^2^	91.8 ± 24.4	91.3 ± 19.6	7.0	0.568	0.940
RV EDVi, mL/m^2^	73.8 ± 13.9	77.2 ± 12.2	8.2	0.010	0.797
RV ESVi, mL/m^2^	25.0 ± 8.9	31.6 ± 8.6	20.5	< 0.001	0.707
RV EF, %	66.6 ± 8.5	59.1 ± 9.5	9.9	< 0.001	0.646
*CMR studies with ischemic heart disease, n = 50*					
LV EDVi, mL/m^2^	80.1 ± 21.7	73.3 ± 19.1	7.3	< 0.001	0.925
LV ESVi, mL/m^2^	36.1 ± 24.3	32.8 ± 20.2	10.8	0.003	0.970
LV EF, %	58.6 ± 15.2	58.0 ± 15.1	6.9	0.449	0.967
LV mass, g/m^2^	67.9 ± 17.3	72.8 ± 17.5	7.5	< 0.001	0.890
RV EDVi, mL/m^2^	78.7 ± 26.1	77.9 ± 25.6	5.4	0.849	0.960
RV ESVi, mL/m^2^	35.5 ± 20.1	39.8 ± 19.5	16.0	< 0.001	0.946
RV EF, %	56.7 ± 11.7	50.0 ± 11.7	11.5	< 0.001	0.837
*CMR studies with congenital heart disease, n = 50*					
LV EDVi, mL/m^2^	86.2 ± 22.8	76.3 ± 21.0	9.2	< 0.001	0.918
LV ESVi, mL/m^2^	31.5 ± 12.6	29.3 ± 12.3	9.6	0.006	0.959
LV EF, %	64.2 ± 7.4	62.3 ± 9.7	5.7	0.203	0.849
LV mass index, g/m^2^	65.6 ± 19.3	69.9 ± 18.2	6.0	0.001	0.928
RV EDVi, mL/m^2^	90.3 ± 18.0	88.5 ± 19.3	7.5	0.885	0.880
RV ESVi, mL/m^2^	37.2 ± 11.8	44.9 ± 13.8	15.4	< 0.001	0.803
RV EF, %	59.2 ± 7.5	49.2 ± 9.9	14.4	< 0.001	0.600

*P-value from Wilcoxon signed-Rank test

Since cases mentioning suboptimal image quality in the clinical reports were excluded, no studies scored 3 when EuroCMR registry image quality criteria were applied [18]. CoVs for all volumetric parameters did not differ significantly when the scores were 0 or 1. When image quality score was 2, variation in LVEDVi (p = 0.001) and LV EF (p = 0.003) increased. Agreement of LV ESVi, LV mass index and RV parameters were not affected by image quality score.

**Fig. 2:**
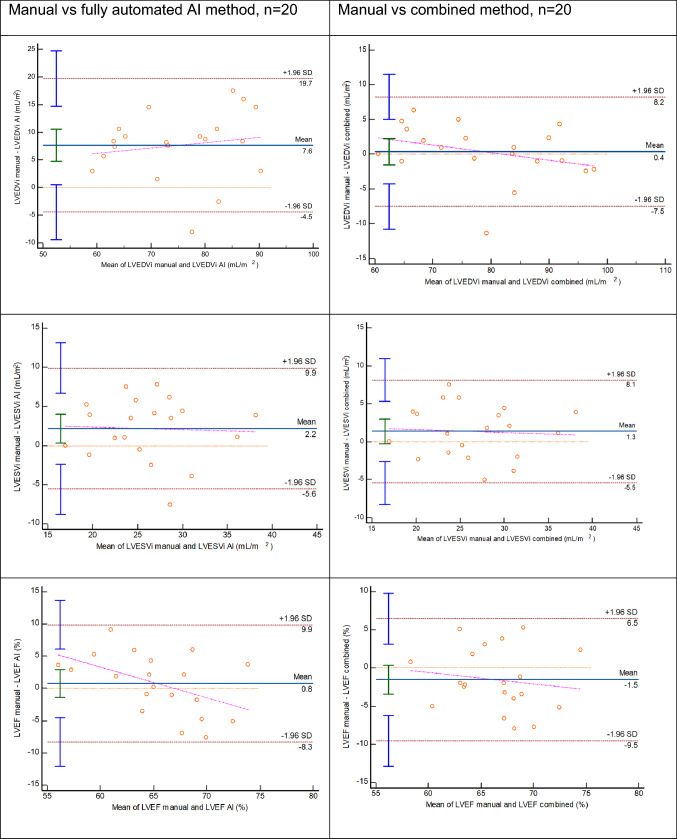

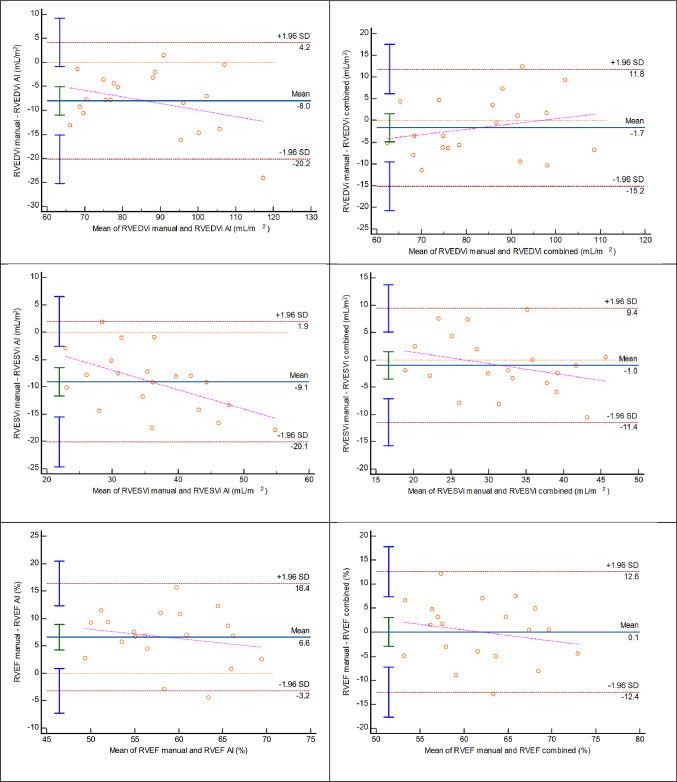
Bland–Altman plots show that agreement substantially improved and mean differences approached zero line when AI contours were manually inspected and adjusted where necessary by the operator (combined method)

With manual adjustment of AI contours within subjects, CoV decreased from 9.1% to 3.5% for LV EDVi; from 12% to 9.7% for LV ESVi; from 5.0% to 4.5% for LV EF; from 8.2% to 5.9% for RV EDVi; from 20.9% to 11.7% for RV ESVi and from 9.9% to 7.1% for RV EF. Bland–Altman plots for this group (n = 20) presented in Fig. 2 show that agreement improved, and mean difference line approached zero for all parameters when combined method was used. There was no statistically significant difference between indexed biventricular volumes, LV mass and biventricular EF when manual values were compared to the combined method output. Single manual expert analysis using CVI^42^ software (n = 20) was compared to manual analysis derived from clinical reports (multiple operators analysed using CMRTools); the agreement was excellent or good and within limits of interobserver variability (Table 3) [20]. Manual expert analysis with CVI^42^ versus fully automated AI analysis followed a trend similar to entire cohort (n = 300) and agreement further improved with combined method.

**Table 3:** Agreement between single manual expert analysis using CVI.^42^ software versus clinically reported manual analysis (CMRTools), fully automated AI analysis and combined method, n = 20

	Manual expert analysis using CVI^42^ software vs. clinically reported manual analysis (CMRTools)		Manual expert analysis using CVI^42^ software vs. fully automated AI analysis		Manual expert analysis using CVI^42^ software vs. combined method*	
	CV %	ICC	CV %	ICC	CV %	ICC
LV EDVi, mL/m^2^	4.0	0.945	5.7	0.911	3.8	0.950
LV ESVi, mL/m^2^	9.6	0.898	11.6	0.931	7.9	0.939
LV EF, %	4.1	0.798	4.7	0.831	4.6	0.749
LV mass index, g/m^2^	3.8	0.969	7.6	0.891	4.7	0.950
RV EDVi, mL/m^2^	7.5	0.848	13.7	0.718	9.0	0.814
RV ESVi, mL/m^2^	15.2	0.795	27.2	0.558	17.1	0.725
RV EF, %	5.5	0.801	11.4	0.605	6.9	0.636

*AI contours inspected and adjusted manually where needed

Manual biventricular volumetric analysis of 20 studies took 250 min 12 s in total whereas the same task was performed in 5 min 48 s using short axis AI myocardial segmentation. Manual analysis per study was timed 718 ± 137 s versus 17(1)s for AI method. AI was approximately 42 × faster than the manual method (p < 0.001). Time spent for visual checking and manual correction of AI contours where necessary with the combined AI and manual method was 247 ± 46 s (n = 20).

### Limitations of AI myocardial segmentation identified on visual assessment

**Fig. 3:**
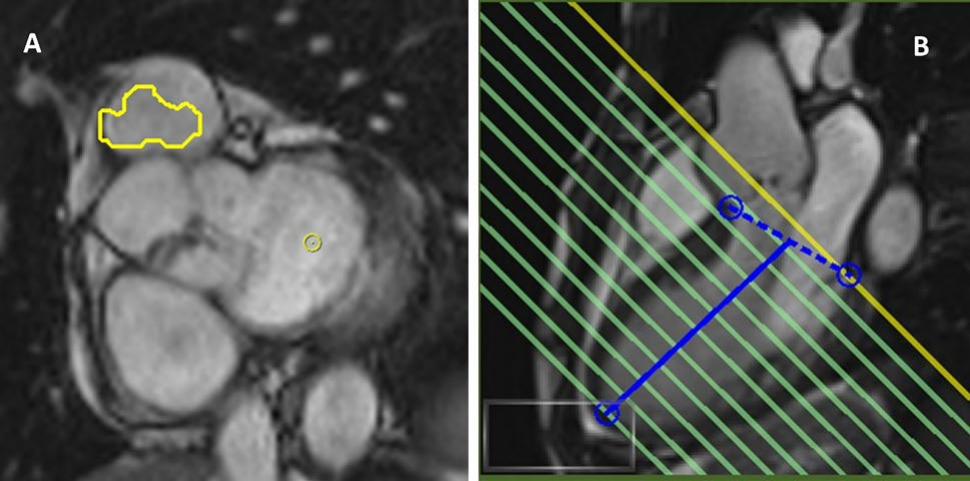
Example of AI analysis at the base of the heart. In Panel **A** there is no red LV endocardial contour, while the cut plane in Panel **B** shows the slice includes a small LV volume in the LVOT region. Mitral valve also appears to be partially open in long axis cine image (Panel **B**) suggestive of inappropriate end diastolic frame selection by AI

**Fig. 4:**
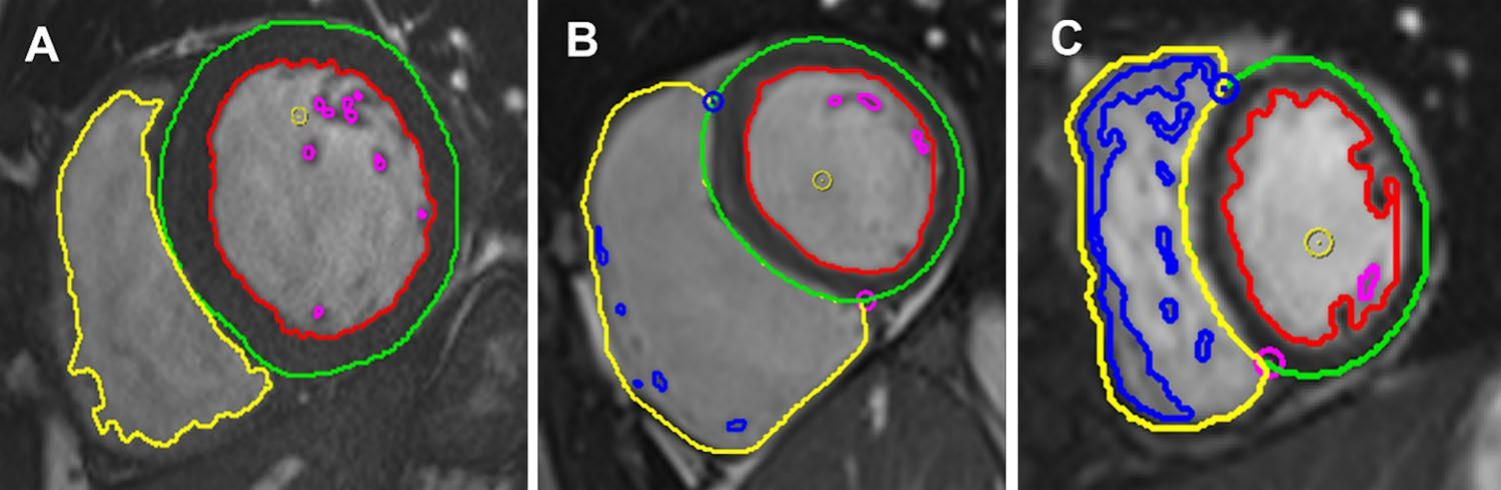
Examples of manual and suboptimal AI myocardial segmentations. Panel **A** shows an ideal example of manual myocardial contouring using the software, please note that the contours exactly delineate the cardiac chamber structures. Panel **B** shows an example of slight underfitting LV endocardial contour which might partly explain underestimation of LV volumes. There is also suboptimal segmentation for LV epicardial, and RV endocardial contours. Panel **C** shows overestimation and suboptimal tracing of RV trabeculations and underfitting of LV endocardial contour in a patient with congenital heart disease

Visual assessment of AI segmentation provided possible explanations for the difference in measurements performed with manual and AI methods. The main observed inaccuracies using AI segmentation were (1) LVOT not included in the volume calculation and hence underestimation of LV volumes, shown in Fig. 3; (2) underfitting of LV endocardial contour which might be another reason for underestimated LV volumes, Fig. 4 Panel B (3) selection of wrong end-diastolic or end-systolic frame for analysis especially when prospective electrocardiographic gating was used for image acquisition Fig. 4; (4) Overestimation as well as suboptimal tracing of RV trabeculations, Fig. 4 Panel C; (5) Errors in excluding RVOT and including RA from RV volumes.

### Survey Responses

Twenty CMR practitioners were invited to complete a survey, 11 out of 13 responders have been practising CMR for more than a year and the remaining two for 6–12 months. Prior to AI clinical accuracy metrics being revealed to the participants, 10 thought that AI segmentation methods could replace manual volumetric analysis in the next 5 years, 8 trusted the AI results and 7 would be confident to use AI analysis results in clinical reports. In terms of efficiency, 11 believed AI would save time and 8 participants thought AI would have a positive impact on their personal wellbeing. When clinical agreement results were presented, 9 (69%) participants reported that results were more reassuring than they had expected. Having seen the performance of AI clinical applications in the department; 9 participants ware keener to use AI and 12 (92%) were looking forward to AI being part of the routine clinical practice.

**Fig. 5:**
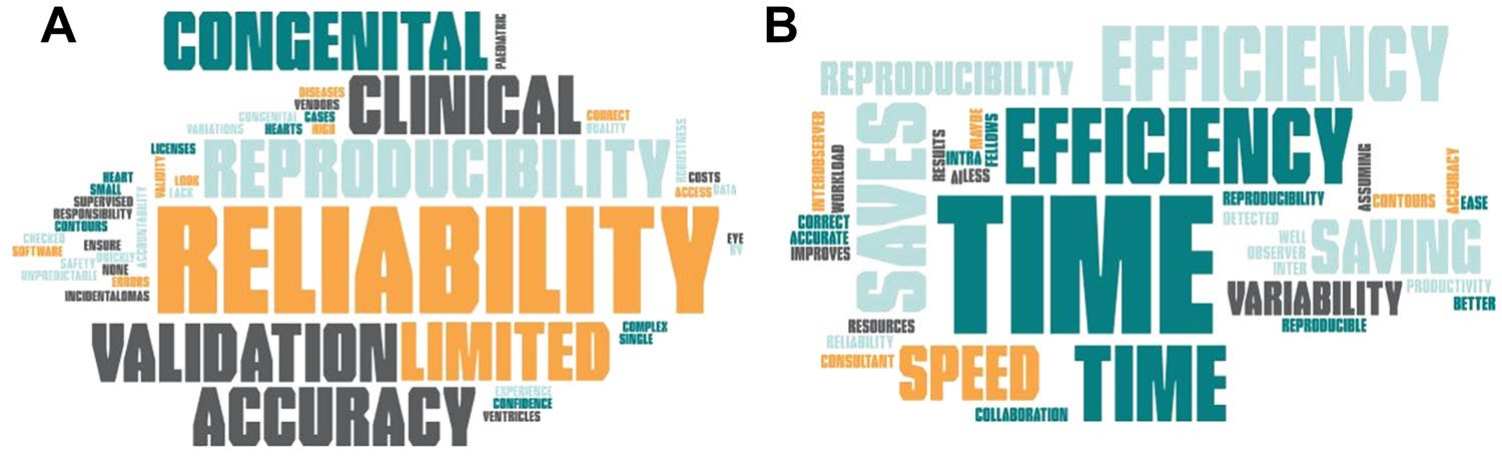
**A** Word cloud on concerns about using AI in clinical practice. **B** Word cloud on potential benefits of adopting the AI volumetric analysis technology

Two open ended questions asked participants to provide words or phrases reflecting their concerns about using AI in clinical practice and potential benefits of adopting the technology. Clinical reproducibility, reliability and validation were main concerns (Fig. 5A). Potential benefits were listed as efficiency, time saving, speed of analysis and better reproducibility (Fig. 5B).

## Discussion

In this study we have shown that AI derived cardiac biventricular volumetric analysis of CMR images produces clinically acceptable results. Manual adjustment of AI derived contours further improves the accuracy of the analysis while still saving significant amount of time (manual analysis time approximately 11.9 min, fully automated AI method 17 s and combined method 3–4 min). The specific software module tested in this work, underestimated indexed LV volumes, LV EF and RV EF and overestimated RV volumes and LV mass index. Agreement for LV parameters was better than RV, however all variations observed were within the range of interobserver agreement reported previously in the literature in non-clinical setting [20]. Therefore, AI analysis results are considered to be clinically applicable. Survey showed that end-user clinicians were open to adopt the specific AI analysis method but concerns about accuracy and clinical validity of results were raised. However, after seeing the agreement results presented in this work, they were keener to use the AI method.

Few studies have previously applied AI myocardial segmentation in large cohorts. Bai et al. trained a CNN model on a large dataset of 4875 scans from UK BioBank cohort. When applied to cases from the UK Biobank cohort the performance was excellent, however in clinical patients the performance was suboptimal but still comparable to human inter-observer variability. Fine-tuning the CNN by retraining with additional clinical cases improved results [21]. Among three tested algorithms, an AI model trained using data from various centers, vendors and pathologies performed better compared to others suggesting feasibility of using the same CNN across multiple centers, vendors, and pathologies [13]. Automated image segmentation yielded precision similar to human analysis suggesting automated segmentation could replace manual analysis [9]. These studies were conducted in research setting using expert analysis as ground truth. In contrast, our work compared clinically reported values with a commercially available AI method. Reproducing the findings in real-life clinical setting provides further reassurance for daily clinical application.

Clinical validation study of another commercial CMR image analysis software (SuiteHEART, NeoSoft, Pewaukee, Wisconsin, USA) compared fully automated biventricular volumetric analysis with manual assessment results in three hundred CMR examinations. In line with our findings, the agreement.

between manual and automated LV assessment was good, while agreement for RV analysis was lower although still comparable to interobserver variability reported in literature. Agreement was the lowest in cases with complex anatomy or reduced image quality [22]. In our study, image quality score of 2 only increased variation in LV EDVi/LVEF values; agreement in RV parameters was not affected. Difference in RV volumes usually originated from inaccurate contouring of RV base resulting in inappropriate inclusion or exclusion of the right atrium and RV outflow tract by AI. Poor image quality usually affects tracing of trabeculations which have a smaller effect on RV volumes. These observations suggest that RV volumetry agreement is not influenced by image quality because the failure in the base of the heart for RV is similar across studies with different image quality. However, unlike previous work we did not include any studies with suboptimal image quality (score 3).

A closer look at the LV EDVi and LV ESVi parameters indicate that AI method systematically underestimated the LV volumes, despite the agreement between two methods being excellent. The same trend of underestimation of LV volumes with AI was also observed in previous reports [9]. LV EDVi CoV was calculated 7.6% (95% CI, 6.0–8.2), which is consistent with Bhuva et al. reporting bias of 6.7% (95% CI, of 4.32–9.37) between neural network and expert analysis [9]. Excluding papillary muscles and trabeculations from the blood pool, which is more complex analysis method, and real-life clinical practice setting of this work might have caused less favourable agreement in our study despite using a later version of the same software. For biventricular volumetric analysis comparing automated versus manual approach, Backhaus et al. have reported ICC values similar to our findings, which are within the limits of human interobserver variability, however when CoVs% were compared (indicator of differences for each individual case), the variation they have reported was clinically significant, whereas performance of the model tested in this work appears to be in closer agreement to manual analysis. For example, for LV ESVi they reported an excellent ICC of 0.96 but CoV of 25% [22]. In this study for the same parameter, we have calculated an ICC of 0.98 and a CoV of 10.9%. Clinicians would desire to have comparable values between analyses for the same subject therefore CoV parameter is more relevant to clinical practice than ICC. Bhuva et al. reported variation of 7.31% (95% CI, 5.4–9.2) for LV ESV using an earlier version of AI model assessed in this work but they compared the model performance with manual analysis in research setting using the same software [9]. The absolute difference in average LV EF between two analysis methods was small, and CoV for LV EF was calculated 6.5% in this study with ICC of 0.95. Bhuva et al. reported 2.95% whereas variation by Backhaus et al. was 10.6% despite an excellent agreement indicator ICC of 0.95 [9, 22]. LV EF is a key parameter in clinical decision-making driving recommendations around therapies such as surgery, intervention, or additional medications [23]. Consensus would not accept a difference in LV EF more than 5% for clinical use [8]. We have achieved this target with manually adjusting AI contours where necessary and CoV improved to 4.5% when the combined method was applied.

There are only a few studies assessing AI myocardial segmentation performance for the right ventricle [10, 21, 22, 24]. In this work, the AI model performed well for RV EDVi with ICC ranging between 0.80–0.96 in different subgroups, whereas outcomes were less favourable for RV ESV and RV EF. These parameters also have higher interobserver variability in clinical practice with ICC reported 0.92 for RV EDV, 0.77 for RV ESV and 0.64 for RV EF in a study with normal subjects [20]. RV at end-systole is the most difficult cardiac region to annotate, even for experienced observers [24]. Since RV EF is a derivative of RVESV, this fact explains the inherent problems of reproducibility for RV. Once again, Backhaus et al. reported similar ICCs compared to this work for RV ESVi and RV EF using AI segmentation, however CoVs showed better performance of the model evaluated in this study with 24.0% variability versus 16.6% in this study for RV ESV and 17.8% variability versus 13.1% in this work for RV EF [22]. Manual adjustment of AI contours (combined method) improved variability in RV ESVi to 11.7% and RV EF to 7.1% in our study.

It is hypothesised that in pathologic conditions heart structures may be more difficult to segment because of high variability in shape or size [24]. Our findings also showed that AI was reliable across variety of cardiovascular pathologies that could potentially distort the usual heart structures and result in uncontrolled variation.

Bernard et al. reported that degenerative AI contours were at the apex or the base at the level of valve planes, however degenerative AI contours were mainly observed at the base of the heart in this study [24]. Visual assessment of the AI contours identified the pitfalls of AI myocardial segmentation providing emphasis for further development of the model. Main issues identified for AI segmentation were poorly defined end-systolic or end-diastolic phase especially on studies with prospective triggering, inaccurate segmentation of LV/RV outflow tract and right atrium at the basal slices, underfitting of LV/RV endocardial contours, overestimation of size as well as suboptimal tracing of RV trabeculations. AI contours score was good or adequate in 83% of cases. Recently, a similar systematic scoring analysis was suggested to determine the clinical acceptability of automated contours focusing on the contours’ clinical utility and aiming to improve clinicians’ confidence in AI and its acceptability in the clinical workflow [25]. AI myocardial segmentation was available using various methods in the commercial software package which we tested. We only tested the method reflecting departmental clinical practice. Volumetric method used in this work included papillary muscles/trabeculations in the myocardium [16, 17]. This is a more complicated analysis since it requires more feature recognition both for humans and AI compared to the alternative method. AI model tested in this work could have performed better if endocardial contour had been selected to be “round” in preferences and biventricular trabeculations would have been excluded from the myocardium [8]. However, we aimed to use AI in order to replicate our routine clinical workflow, not the other way round. Adapting the analysis method to a more simplified version with the sole intention to increase AI accuracy has potential to jeopardize trust in the capabilities of technology.

Our observed manual analysis time of average 11.9 min, ranging between 8 and 17 min, is comparable to previous reports [20–22]. AI analysis time was 17 s and adjusting the AI segmentation when needed took 4.1 min (combined method) further optimising the agreement with clinically reported values. In 2018 a total of 114,967 CMR studies were performed in UK, therefore clinical application of AI analysis has huge potential to save approximately 22,388 clinician hours for the fully automated AI method and 15,042 clinician hours per year with the combined method [26].

## Limitations

This work compared the AI performance with manual volumetric and functional analysis in routine clinical setting, however reliability of reported data is dependent on the operator. Although all reporting clinicians were experts, another software package (CMRtools) was used to analyse clinical volumes. This fact might have also contributed the variation observed. To address this limitation, in a subset of cases (n = 20) manual analysis using CVI^42^ software was compared to manual analysis derived from clinical reports, the agreement was excellent/good and within limits of interobserver variability. When manual CVI^42^ volumes were compared to fully automated AI analysis, results reflected a similar trend to the entire cohort suggesting that differences between vendors and multiple experienced operators are minor and likely negligible in the clinical setting. A large sample size was chosen with various pathologies but still the results may not be generalisable for the entire spectrum of cardiac pathologies other than covered in this work. Of note, complex congenital cardiac diseases with single or complex biventricular physiology have not been included into this study. Finally, AI is an ever-developing field, improved models with potentially better performance than tested in this work were developed during the study period. A new version (5.13) of the evaluated software with an enhanced AI module was launched when data collection for this work was already completed [27]. Therefore, current AI performance of the product and other AI models discussed might be different, likely better, than presented in this work.

## Supplementary Information

Below is the link to the electronic supplementary material.

Supplementary file1 (PDF 14 kb)

## Data Availability

The data underlying this article will be shared on reasonable request to the corresponding author.
