# Supplementary material for: Performance of artificial intelligence for biventricular cardiovascular magnetic resonance volumetric analysis in the clinical setting
Source: Int J Cardiovasc Imaging. 2022 Jun 29;38(11):2413–24. doi: 10.1007/s10554-022-02649-1 (PMC9700578; doi:10.1007/s10554-022-02649-1)
Supplement: Supplementary file 1 — Supplementary file1 (PDF 14 kb) [file 10554_2022_2649_MOESM1_ESM.pdf]

Appendix 1
Survey

1. How long have you been practicing CMR?
   0-3 months   3-6months   6-12months   >1 year

2. How many CMR volumetric analyses have you performed in the past 12 months?
   0-100   100-300   300-500   >500

3. I feel confident about my volumetric analysis results and I am rarely challenged on the validity of results by my seniors.
1)Strongly disagree
2)Somewhat disagree
3)Neither agree nor disagree
4)Somewhat agree
5)Strongly agree

4. In my opinion, AI segmentation methods in its current form can replace manual CMR volumetric analysis in the next 5 years.
1)Strongly disagree
2)Somewhat disagree
3)Neither agree nor disagree
4)Somewhat agree
5)Strongly agree

5. I trust the results obtained by AI contorting for volumetric CMR analysis.
1)Strongly disagree
2)Somewhat disagree
3)Neither agree nor disagree
4)Somewhat agree
5)Strongly agree

6. I would be confident to use the values provided by AI analysis on my clinical reports.
1)Strongly disagree
2)Somewhat disagree
3)Neither agree nor disagree
4)Somewhat agree
5)Strongly agree

7. I believe replacing the manual method with AI will save considerable amount of time for me.
1)Strongly disagree
2)Somewhat disagree
3)Neither agree nor disagree
4)Somewhat agree
5)Strongly agree

8. I believe AI will have positive impact on my work efficiency and personal wellbeing within the next 5 years.

1)Strongly disagree
2)Somewhat disagree
3)Neither agree nor disagree
4)Somewhat agree
5)Strongly agree

9. We have tested the agreement between AI and manual analysis on clinical reports for 300 cases across various pathology groups. ICC results showed that there was good agreement for LV parameters and acceptable agreement between RV parameters tabulated below. These results are more reassuring than I had expected.
Table of agreement analysis results for all 5 groups.
1)Strongly disagree
2)Somewhat disagree
3)Neither agree nor disagree
4)Somewhat agree
5)Strongly agree

10. Having seen agreement test results I am keener to use AI methods in clinical practice.
1)Strongly disagree
2)Somewhat disagree
3)Neither agree nor disagree
4)Somewhat agree
5)Strongly agree

11. I look forward to AI being part of routine clinical practice.
1)Strongly disagree
2)Somewhat disagree
3)Neither agree nor disagree
4)Somewhat agree
5)Strongly agree

12. Could you provide three words representing your concerns about using AI in routine clinical practice?

13. Could you provide three words representing the possible benefits of using AI in routine clinical practice?
